# Ultralow concentrations of curcumin as a structural modifier in chitosan coatings for eco-friendly postharvest preservation of strawberries

**DOI:** 10.1007/s10068-026-02245-2

**Published:** 2026-07-17

**Authors:** Henrique Soares Silva, Anna Paulla Ferreira Araújo Zanoli, Hellen Franciane Gonçalves Barbosa, Rafael de Oliveira Pedro

**Affiliations:** Department of Exact and Earth Sciences, Academic Unit of Ituiutaba, State University of Minas Gerais (UEMG), Rua Vereador Geraldo Moisés da Silva, s/n, Bairro Universitário, Ituiutaba, MG 38302-192 Brazil

**Keywords:** Chitosan edible coating, Curcumin, Postharvest quality, Strawberry preservation, Biodegradable packaging

## Abstract

This study evaluated the impact of low concentrations of curcumin (2.5–20 µM) on chitosan edible coatings for strawberry preservation. Films obtained by solvent casting were characterized by ATR-FTIR spectroscopy, thickness, moisture content, water vapor permeability, water solubility, and soil degradability. The results indicate that curcumin reduces moisture, solubility, and biodegradability, suggesting the formation of a more cohesive matrix. Furthermore, the spectroscopy results demonstrated that curcumin interacts with chitosan chains through hydrogen bonds without cross-linking. To test the protective effects, strawberries were coated and stored at 20 °C for 7 days. Data on weight loss, visual decay, firmness, soluble solids content, and color change (ΔE) confirmed that chitosan-curcumin (CS-Cur) formulations were superior to uncoated and neat chitosan controls. Considering that even at low concentrations, curcumin acts as a structural modifier and bioactive agent, CS-Cur coatings have potential as an ecological package for fresh fruit.

**Supplementary Information:**

The online version contains supplementary material available at https://doi.org/10.1007/s10068-026-02245-2.

## Introduction

Strawberries (*Fragaria* × *ananassa*) are widely consumed because of their distinctive flavor, nutritional value, and excellent antioxidant capacity. However, their high susceptibility to rapid moisture loss, fungal deterioration, and mechanical damage results in a very short postharvest shelf life, causing significant economic losses (Ali et al., 2022; Gigante et al., 2025). To minimize these losses, several strategies have been employed, with traditional plastic packaging being one of the most commonly used alternatives. However, these synthetic packages are considered significant environmental problems because of their difficulty in biodegradation. To reduce postharvest losses and minimize the use of synthetic packaging, greater attention has been given to sustainable, biodegradable, and active packaging films (Choi et al., 2023; Nguyen et al., 2025; Sharma et al., 2025).

One strategy for obtaining biodegradable packaging is to use edible coatings based on biopolymers such as chitosan. This polycationic polymer is derived from chitin and is widely used for various purposes because of its biocompatibility, biodegradability, antimicrobial activity, and film-forming properties (Duan et al., 2019; Saberi Riseh et al., 2024). Chitosan is capable of forming a semipermeable barrier on the surface of fruits, which restricts transpiration and senescence. Despite these characteristics, these coatings are highly hydrophilic and have limited antioxidant activity, which may restrict their protective capacity.

To improve the protective properties of chitosan films, many studies have focused on the addition of bioactive compounds, such as curcumin (Cur), a natural polyphenolic compound recognized for its antioxidant and antimicrobial activities (Avendaño-Briseño et al., 2025; El-Saadony et al., 2025; Hussain et al., 2022). The polyphenolic structure of curcumin allows noncovalent interactions with the functional groups of chitosan, particularly hydrogen bonds between its hydroxyl and carbonyl groups and the amine and hydroxyl groups of chitosan. These interactions can modulate chain packing, alter hydrophilicity, and improve the cohesive integrity of the matrix without requiring chemical modification of the polymer. Although previous studies have investigated chitosan-curcumin blends, they frequently rely on crude extracts (Rachtanapun et al., 2021), high curcumin concentrations, or complex stabilization methods such as chemical cross-linking (Kalaycıoğlu et al., 2017) and photodynamic inactivation (PDI) (Wang et al., 2022). While effective, coatings with high concentrations of curcumin may have compromised mechanical integrity, in addition potentially to altering the sensory properties of the food. Moreover, complex physicochemical treatments may be a barrier to industrial scalability.

It is not yet clear whether the structural-modifying effect of curcumin on the chitosan matrix is preserved at micromolar concentrations, as this concentration range has not been systematically explored in the literature. Furthermore, most research remains restricted to in vitro characteristics. The use of highly purified curcumin at ultralow (micromolar) concentrations as a simple structural modifier and its subsequent in situ performance on highly perishable matrices remain poorly explored.

Therefore, the objective of this study was to develop and characterize simple, eco-friendly chitosan-based films incorporating micromolar concentrations of curcumin (2.5–20 µM) and evaluate their physicochemical, structural, and biodegradability properties. Additionally, this study aimed to assess the in situ effectiveness of these composite coatings in extending the shelf-life and preserving the postharvest quality (weight loss, visual decay, firmness, color, and soluble solids) of fresh strawberries during ambient storage, bridging the gap between in vitro barrier properties and real-world applications.

## Materials and methods

### Materials

Chitosan (viscosity of 20–800 mPa/s) was acquired from Êxodo Cieintífica (Sumaré, São Paulo, Brazil) and used without further purification. Curcumin from *Curcuma longa* (Turmeric) was purchased from Merck (Darmstadt, Germany). Hydrochloric acid, acetic acid, ethanol, sodium hydroxide, deuterated water, and deuterium chloride were purchased from Sigma‒Aldrich (São Paulo, Brazil). The reagents used were of analytical grade and were used as received. All aqueous solutions were prepared with ultrapure water (resistivity > 18.2 MΩ cm) from a Milli-Q system (Merck Millipore, Darmstadt, Germany).

Fresh strawberries with a uniform appearance (i.e., size, color, and shape), without signs of physical deterioration or fungal infection, were randomly selected from a local market. The acquired samples were washed with distilled water and disinfected by immersion in a sodium hypochlorite solution (125 ppm active chlorine) for five minutes. After disinfection, the strawberries were air-dried until the water completely evaporated.

### Chitosan characterization methods

#### Determination of the average degree of deacetylation

The average degree of deacetylation $\left(\overline{DD }\right)$ of chitosan was determined via potentiometric titration according to the literature (Tolaimate et al., 2000). For this purpose, 40 mg of chitosan was first dried in an oven at 50 °C until a constant mass value was reached and then dissolved in 10 mL of standardized hydrochloric acid (0.091 mol L^−1^). The resulting solution was then titrated with a standardized sodium hydroxide solution (0.099 mol L^−1^), and the pH was monitored via a pH meter during the titration process (Oliveira Pedro et al., 2013).

The $\overline{DD }$ was also obtained via the ^1^H NMR technique. For this purpose, approximately 20 mg of chitosan sample was added to 1 mL of deuterated water containing 10 μL of deuterium chloride. The dispersions were stirred until complete solubilization and subsequently transferred to 5 mm NMR tubes. The ^1^H NMR spectra were acquired at 70 °C on a Fourier300 UltraShield® spectrometer equipped with a 7.05-T magnet and a 300 MHz dual direct detection probe (Bruker, Madison, USA).

### Characterization of the film samples

#### Preparation of coating solutions

A chitosan solution (1% w/v, 250 mL) for film fabrication was prepared by dispersing the biopolymer in a glacial acetic acid solution (1% v/v) according to the method proposed in the literature (Choi et al., 2023). The mixture was then stirred for 12 h. The plasticizer glycerol (0.5% w/v) was added to the film-forming solution to improve the handling of the films. Subsequently, 5 mL of ethanolic curcumin solution was added to the chitosan solution to yield final curcumin concentrations of 2.5, 5, 10, and 20 µM. To isolate the effect of curcumin and rule out any solvent-induced microstructural changes, the control group (neat chitosan) received an equivalent volume of ethanol.

#### Preparation of films for physicochemical characterization (solvent casting)

Chitosan-based films, with (CS-Cur) or without curcumin (CS), were prepared as standalone films specifically for physicochemical characterization. Briefly, 20 mL of the film-forming solution was poured into polystyrene Petri dishes (90 × 15 mm) (Du et al., 2025). The Petri dishes were dried in an oven at 50 °C for 48 h in the dark. After drying, the films were peeled from the Petri dishes and conditioned at 25 ± 2 °C and 50 ± 5% relative humidity for 48 h prior to physicochemical analysis.

#### Determination of film thickness and moisture content

The film thickness was measured at five random positions via a digital micrometer (Digimess, model 110284NEW; 0.001 mm resolution). The mean thickness was used for subsequent analyses, as is commonly reported for cast biopolymer films (Shiekh et al., 2022).

The film moisture content was determined via a halogen lamp moisture analyzer (Gehaka, model IV3050). Three independent film samples (area 4 cm^2^) were analyzed. Measurements were performed at 105 °C for 10 min, and the moisture content was expressed as a percentage (%) (Pamlényi et al., 2025).

#### ATR-FTIR analysis of the films

Attenuated total reflectance Fourier transform infrared (ATR–FTIR) spectroscopy was used to assess chemical interactions and structural changes in the polymeric film matrices. Spectra were collected via a Cary 630 FTIR spectrometer (Agilent Technologies Inc., Santa Clara, CA, USA) over the 4000–600 cm⁻^1^ wavenumber range, with 64 scans and a 4 cm⁻^1^ resolution.

#### Water vapor permeability

The water vapor permeability (WVP) of the films was determined via the dry-cup method according to ASTM-E96 (cup method). The films were sealed over a circular opening in perforated glass lids (the exposed area A defined by the opening diameter), and an O-ring was used to prevent edge leakage. Each cup was filled with 10 g of anhydrous calcium chloride (previously oven-dried) to provide ${RH}_{int}\approx 0$. The assemblies were placed in a sealed desiccator at 25 °C containing a saturated salt solution to maintain ${RH}_{ext}\approx 0.75$. The cups were weighed (± 0.0001 g) at predetermined intervals, and the mass gain (m, g) was plotted as a function of time. The term w/t corresponds to the water vapor transmission rate in mass units and was obtained as the slope (dm/dt) of the linear regression of mass gain versus time under steady-state conditions (g·day⁻^1^). The water vapor transmission rate was obtained from the slope of the linear region of mass gain versus time. The water vapor transmission rate was calculated as:

1 $$WVP=\frac{(w/t)x}{A\Delta P}$$

where x is the film thickness (mm), A is the exposed area (m^2^), and ΔP is the water vapor partial pressure difference across the film (kPa), which is calculated as:

2 $$\Delta P={P}_{sat}(25^\circ{\rm C} )({R}_{1}-{R}_{2})$$

where ${P}_{sat}=3.166 \mathrm{k}\mathrm{P}\mathrm{a}$, ${R}_{1}=0.75$ (external RH) and ${R}_{2}\approx 0.00$ (internal RH, dry-cup).

## Biodegradability

### Water solubility

The water solubilities of the films were determined according to methods described in the literature (Kaya et al., 2018), with slight modifications. Film samples (2 × 1 cm) were cut and weighed to obtain the initial dry mass (Wᵢ). Each sample was then immersed in 20 mL of distilled water in a 25 mL beaker and kept at room temperature for 48 h (Shiryanpour et al., 2025). After immersion, the remaining film residues were carefully recovered and dried in an oven at 60 °C until a constant mass was reached. The dried residues were weighed to determine the final dry mass (W_f_). Measurements were performed using three independent samples for each film formulation (n = 3). The results are expressed as the mean value of three measurements, and the following equation was used for the calculation:

3 $$\mathrm{S}\mathrm{o}\mathrm{l}\mathrm{u}\mathrm{b}\mathrm{i}\mathrm{l}\mathrm{i}\mathrm{t}\mathrm{y} \left(\%\right)=\left[\frac{\left({W}_{i}-{W}_{f}\right)}{{W}_{i}}\right]\times 100$$

### Soil degradability

The soil degradability of the films was assessed according to a previously described method (Kaya et al., 2018). Dry film samples (2 × 1 cm) were first weighed to obtain the initial dry mass (Wᵢ) and then placed in containers filled with natural Latosol soil (collected from Ituiutaba, Brazil, sieved through a 10 mesh). To establish moisture conditions favorable for biodegradation, 10 mL of water was added to each container.

The samples were kept in contact with the soil for 31 days under ambient conditions, without the addition of exogenous microorganisms, with the intention of preserving the original microflora of the soil. Finally, the films were carefully recovered, gently cleaned with a soft brush to remove adhered soil particles, dried in an oven at 60 °C until a constant mass was reached, and weighed again to determine the final dry mass (W_f_).

Each formulation was analyzed in triplicate (n = 3). The soil degradability was expressed as the percentage mass loss, which was calculated according to the following equation:

4 $$\mathrm{S}\mathrm{o}\mathrm{i}\mathrm{l} \mathrm{d}\mathrm{e}\mathrm{g}\mathrm{r}\mathrm{a}\mathrm{d}\mathrm{a}\mathrm{b}\mathrm{i}\mathrm{l}\mathrm{i}\mathrm{t}\mathrm{y} \left(\%\right)=\left[\frac{\left({W}_{i}-{W}_{f}\right)}{{W}_{i}}\right]\times 100$$

where Wᵢ and W_f_ are the initial and final dry masses of the film samples, respectively.

### Application of coatings on fruits

The prepared coating formulations were applied to strawberries by immersion (dipping) in the corresponding film-forming solutions. Each fruit was immersed individually for 2 min, removed, and allowed to dry at room temperature. A total of 54 strawberries from three independent batches were randomly allocated into six experimental groups: Control (uncoated), CS, CS-Cur-2.5, CS-Cur-5, CS-Cur-10, and CS-Cur-20. Each treatment consisted of 9 fruits, which were divided into 3 biological replicates of 3 fruits each. This randomized block design was used to minimize the natural biological variability among individual fruits. After coating, the fruits were stored at 20 ± 1 °C and 75 ± 5% relative humidity (RH) for 7 days to simulate ambient storage conditions (González-Cuello et al., 2024).

### Weight loss measurement

The weight loss (WL, %) of fruits coated with chitosan-based films was monitored during storage. Individual fruit weights were recorded at predetermined time intervals and compared with those of the control group (uncoated fruits). WL was expressed as a percentage of the initial weight and calculated according to the following equation (Pervez et al., 2026):

5 $$WL\left(\%\right)=\frac{{W}_{0}-{W}_{t}}{{W}_{0}}\times 100$$

where ${W}_{0}$ is the initial fruit weight (day 0) and ${W}_{t}$ is the fruit weight at time t (sampling day).

### Visual decay

Visual decay was assessed by daily visual inspection of the strawberries, as described in the literature (Khan et al., 2019; Peretto et al., 2017). Fruits were classified as decayed when any visible lesion indicative of deterioration was observed, including brown spotting or signs of microbial infection. The results are expressed as the percentage of decayed fruits relative to the total number of fruits evaluated in each treatment.

### Soluble solids content

The soluble solids content (SSC) was measured at day 7 of storage via a digital handheld refractometer (0–85°Brix; Milwaukee Instruments, model MA871) calibrated with distilled water at 20 °C. For each sample, strawberries were manually squeezed, and an aliquot (1 mL) of juice was placed on the prism to obtain the SSC reading, following procedures reported in the literature (Su et al., 2021). The results were recorded as °Brix per sample.

### Fruit color parameter determination (CIELab) and total color difference (ΔE)

The CIELab color space was used to monitor the color changes of the stored strawberries. Images were acquired via a smartphone camera (Samsung S24 +) and the Color Grab application. A closed photographic box, composed of a white background and diffuse lighting, was used to capture the images. The distance between the camera and the sample was kept constant.

In the CIELab system, L* represents lightness (0 = black; 100 = white), whereas a* describes the green (−) to red (+) axis and b* the blue (−) to yellow (+) (Gigante et al., 2025). The overall color change during storage was quantified by the total color difference (ΔE*), which was calculated relative to the initial measurement (day 0) according to:

6 $$\Delta E=\sqrt{{\Delta L}^{2}+{\Delta a}^{2}+{\Delta b}^{2}}$$

where ΔL*, Δa*, and Δb* are the differences in lightness (L*), red–green coordinate (a*), and yellow–blue coordinate (b*), respectively, calculated for each fruit as the value on day 7 minus the corresponding baseline value on day 0.

### Firmness

Strawberry firmness was measured at the fruit core region on day 7 of storage, as described in the literature (Gigante et al., 2025). Fruits were halved, and firmness was determined via a digital penetrometer (PTR-500, Instruterm) fitted with an 11 mm diameter probe. The instrument operates over a measurement range of 0.2–15 kgf/cm^2^. Five fruits per treatment were analyzed, and one measurement was taken from the core region of each fruit half (totaling two readings per fruit), with the results expressed as the mean value per fruit.

### Statistical analysis

The experiment was conducted in accordance with a completely randomized design. Statistical analyses were performed in OriginPro 2026 (OriginLab Corporation, USA). The data were analyzed by analysis of variance (ANOVA), and when significant effects were detected (p < 0.05), mean comparisons were carried out via Tukey’s HSD test (α = 0.05). The results are expressed as the means ± SDs of three independent replicates (n = 3) for each sample. ANOVA assumptions were checked by residual diagnostics (normality and homoscedasticity).

## Results and discussion

### Characterization of chitosan

**Fig. 1:**
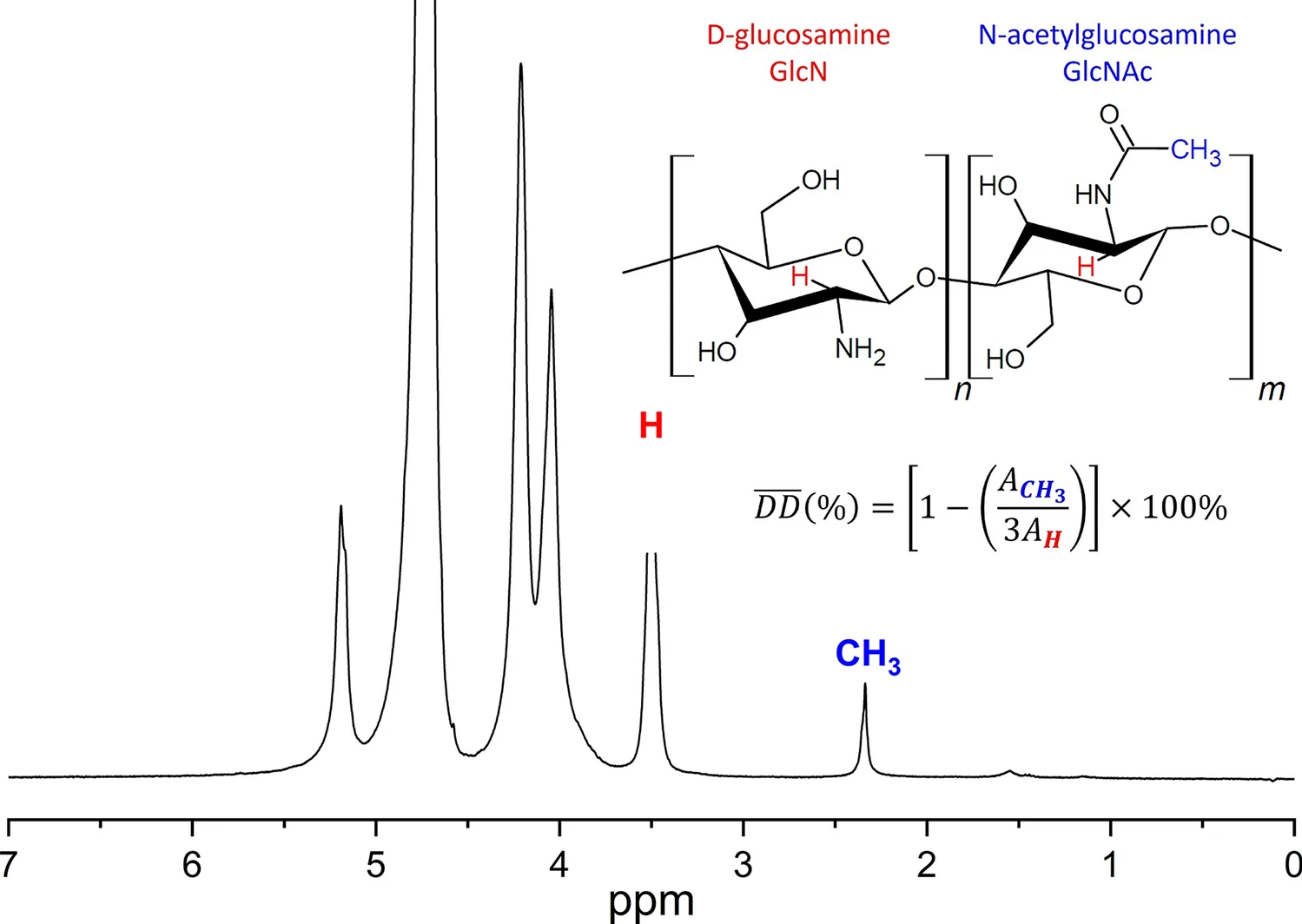
^1^H-NMR spectra of chitosan dissolved in D2O/DCl

The average degree of deacetylation $\left(\overline{DD }\right)$ of chitosan was determined via ^1^H NMR using D₂O/HCl as the solvent system. Spectra were acquired at 70 °C to reduce the solution viscosity and improve peak resolution, thereby minimizing the line broadening effects associated with polymer acetylation and acid hydrolysis. Figure 1 presents a representative ^1^H NMR spectrum of the deacetylated chitosan.

The average DD was calculated from the ratio of the integrated peak areas assigned to (i) the acetamidomethyl protons (–NHCOCH₃) at δ ≈ 2.3 ppm and (ii) the H-2 proton of the glucosamine ring at δ ≈ 3.5 ppm, as previously described (Oliveira Pedro et al., 2016). The equation shown in Fig. 1 was used, yielding a DD of 91.4%. The $\overline{DD }$ was additionally determined by potentiometric titration, yielding $\overline{DD }$ = 95.6%.

### Film thickness, moisture content and water vapor permeability

Table 1 summarizes the physicochemical properties of the solvent-cast CS films. Film thickness was significantly affected by the addition of curcumin (p < 0.05). The neat CS film exhibited the greatest thickness, whereas the incorporation of curcumin led to thinner films, with a nonmonotonic dependence on the curcumin concentration. Even at the lowest level (2.5 µM), curcumin markedly reduced the thickness. This behavior indicates that at ultralow concentrations, curcumin molecules act as highly efficient structural bridges, maximizing intermolecular hydrogen bonding and facilitating a highly compact chitosan chain packing without introducing steric hindrance. At intermediate doses (5–10 µM), the thickness slightly increased relative to the 2.5 µM formulation. This is likely because the accumulation of bulky polyphenolic rings begins to introduce steric hindrance, slightly pushing the polymer chains apart (Li et al., 2023). Finally, the further decrease in thickness observed at 20 µM may be attributed to the onset of curcumin self-aggregation, or to a secondary reorganization of the polymeric network during solvent evaporation that promotes further compaction of the matrix (Liu et al., 2016). Taken together, these observations are consistent with a concentration-dependent transition in the mode of curcumin-chitosan interaction, where chain compaction is most efficiently achieved at the lowest concentration, where molecular dispersion is maximized.

**Table 1:** Formulation composition, thickness, moisture content, and water vapor permeability (WVP) of solvent-cast chitosan (CS) films containing different curcumin concentrations

Formulation	Curcumin (µM)	Thickness (mm)	Moisture content (%)	WVP (g·mm·m^−2^·day^−1^·kPa^−1^)
CS	–	0.194 ± 0.05^a^	8.02 ± 0.14^a^	0.121 ± 0.002^b^
CS-Cur-2.5	2.5	0.123 ± 0.02^d^	7.71 ± 0.12^a^	0.231 ± 0.021^a^
CS-Cur-5	5.0	0.168 ± 0.02^b^	7.34 ± 0.07^b^	0.253 ± 0.012^a^
CS-Cur-10	10	0.165 ± 0.09^b^	7.01 ± 0.09^c^	0.256 ± 0.013^a^
CS-Cur-20	20	0.140 ± 0.04^c^	6.64 ± 0.10^d^	0.264 ± 0.005^a^

The values are expressed as the means ± standard deviations (n = 5). Different lowercase letters within the same column indicate significant differences among formulations (one-way ANOVA followed by Tukey’s post hoc test, p < 0.05)

These findings contrast with those of (Rachtanapun et al., 2021), who reported no significant thickness changes in chitosan films prepared with alcoholic curcumin extract at higher nominal curcuminoid concentrations (217–1085 µM). Unlike that study, the present work employed a system composed of purified chitosan and analytical-grade curcumin. This system has a controlled composition, which allows us to infer that the observed physicochemical alterations can be attributed to the curcumin-chitosan interaction. Thus, the system in the present study does not interfere with cosolutes or other constituents typically present in crude extracts.

In parallel, the moisture content decreased with increasing curcumin concentration, decreasing from 8.02 ± 0.14% (control) to 6.64 ± 0.10% at 20 µM. This behavior points to a reduced water affinity, likely caused by denser chain packing that restricts access to hydrophilic binding sites.

Interestingly, water vapor permeability (WVP) significantly increased for all the curcumin-containing films compared with that of the neat CS control, although WVP remained stable across the different curcumin doses (p > 0.05). The data on the simultaneous reduction in thickness and moisture content associated with an increase in WVP suggest that curcumin decreases water sorption and increases vapor diffusion through the matrix. This behavior may occur due to an increase in free volume or microstructural heterogeneity (Łupina et al., 2019; Wang et al., 2022).

It should be noted that tensile strength and elongation at break are properties typically assessed in self-standing films, where the material itself bears the mechanical load. In the coating application scenario investigated here, where the material is applied as a thin layer directly onto the fruit surface, the functional performance is more directly governed by parameters such as water vapor permeability, surface wettability, and the antimicrobial and antioxidant activity of the coating, all of which were characterized. Nonetheless, the mechanical characterization of curcumin-chitosan films is well documented in the literature. For instance, recent studies have demonstrated that curcumin incorporation significantly increases both tensile strength and elongation at break in chitosan films, a behavior attributed to improved interfacial adhesion mediated by strong intermolecular hydrogen bonding (Madian et al., 2023). Similar mechanical improvements have also been reported in chitosan matrices incorporated with crude turmeric extracts (Kalaycıoğlu et al., 2017). Furthermore, Wang et al. (2022) reported consistent reinforcing effects in ternary composite films (GEL/CS/Cur), further corroborating that curcumin enhances structural integrity through hydrogen bonding interactions.

### ATR-FTIR analysis of the films

**Fig. 2:**
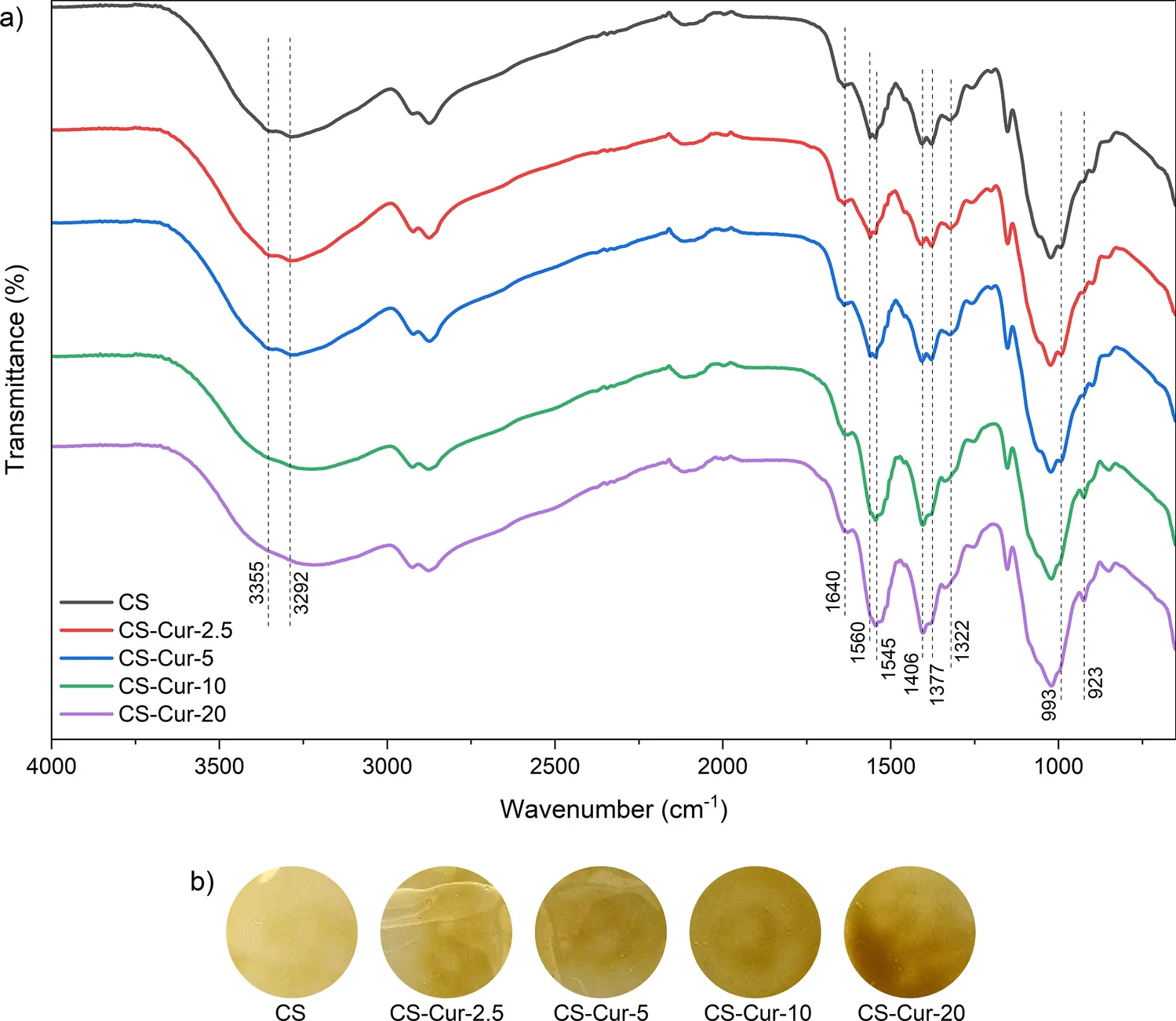
**a** ATR–FTIR spectra of chitosan (CS) films and chitosan films containing increasing concentrations of curcumin (2.5–20 µM) in the 4000–650 cm⁻^1^ range. The dashed lines indicate the selected bands used for peak assignment. **b** Visual appearance of the corresponding standalone films

Although the concentration of curcumin incorporated into the films was low (2.5–20 µM), systematic changes can be observed in the FTIR spectra. As shown in Fig. 2, in the O–H and N–H stretching regions at 3355 and 3292 cm⁻^1^, respectively, (Cheng et al., 2020) slightly shifted and progressively changed in the band profile with increasing curcumin concentration. This behavior is consistent with the formation of additional hydrogen bonds between the hydroxyl and carbonyl groups of curcumin and the amine and hydroxyl groups of chitosan (Lu et al., 2025).

Subtle changes in band shape and relative intensity are observed in the amide I region (1640 cm⁻^1^), which can be attributed to modifications in the hydrogen-bonding environment of chitosan carbonyls (residual N-acetylated units) and to spectral overlap with curcumin vibrations in this region. The amide II deformations (1560 and 1545 cm⁻^1^) slightly shifted toward lower wavenumbers and changed in profile, suggesting that the chitosan amine groups (–NH₂/–NH₃⁺) interact with curcumin, most likely through hydrogen bonding or electrostatic contributions, rather than through covalent bond formation, as suggested by several authors (Kalaycıoğlu et al., 2017; Wang et al., 2019; Zhang et al., 2020). Changes are also observed in the band profiles at 1406 and 1377 cm⁻^1^, assigned to CH₃ and CH₂ deformations. These alterations indicate local rearrangements in the chitosan polymer chain packing and conformation to accommodate the curcumin molecules within the matrix.

The profile of the amide III band (1322 cm⁻^1^) slightly changes, which is consistent with the involvement of C–N and C–O groups in intermolecular interactions with the bioactive compound. Finally, modifications in the structural microenvironment and chain organization of the chitosan matrix induced by curcumin are evidenced by slight changes in the fingerprint region bands (993 and 923 cm⁻^1^), which are sensitive to polysaccharide backbone conformation and glycosidic vibrations.

### Water solubility and soil degradability

**Fig. 3:**
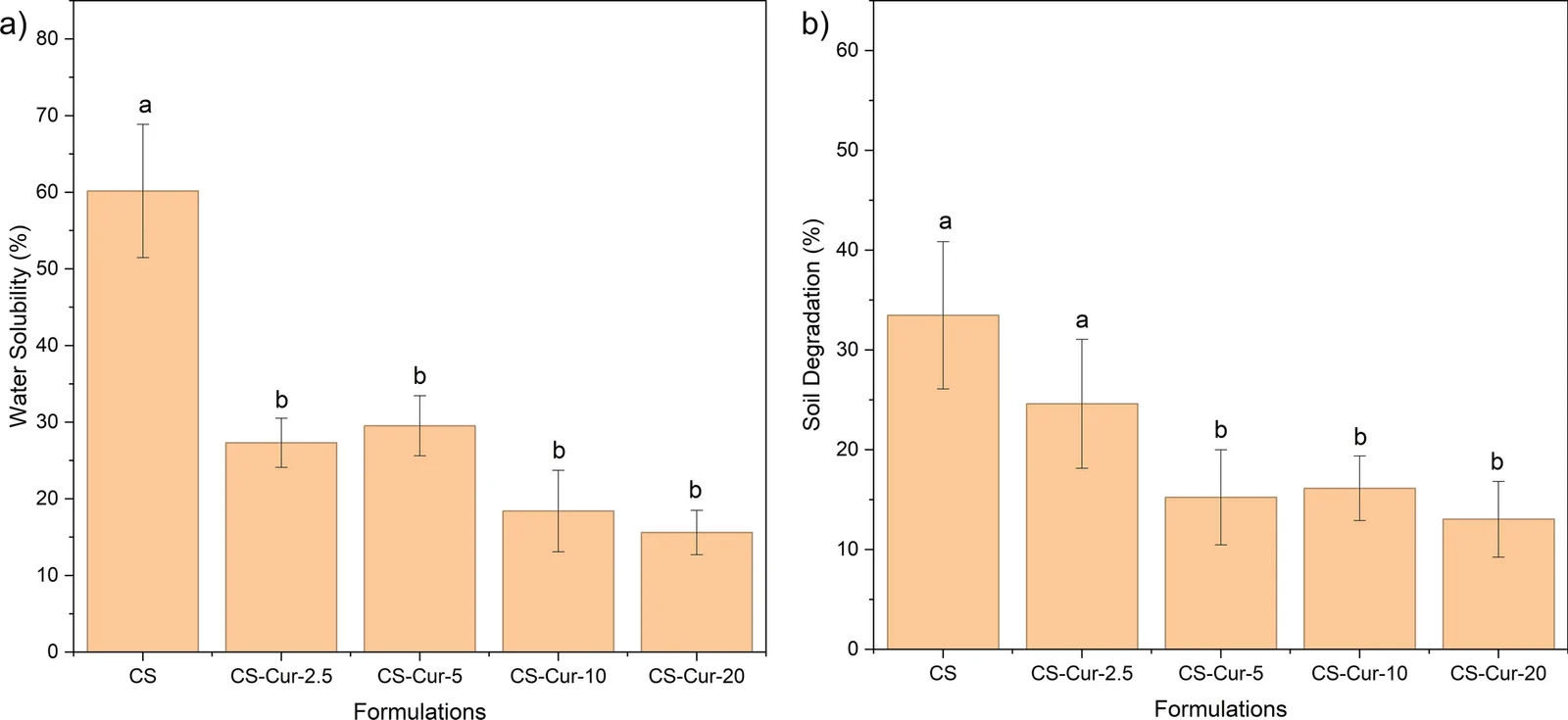
Water solubility and soil degradability of chitosan (CS) films and curcumin-loaded films (CS-Cur; 2.5–20 µM). **a** Water solubility (%) determined as mass loss after immersion in distilled water for 48 h at room temperature. **b** Soil degradability (%) was determined as mass loss after 31 days of incubation in natural Latosol soil under ambient conditions. The bars represent the means ± standard deviations (n = 3). Different letters above the bars indicate significant differences among formulations (one-way ANOVA followed by Tukey’s post hoc test, p < 0.05)

The incorporation of curcumin into chitosan films significantly affected both water solubility and soil degradability, as shown in Fig. 3. Neat chitosan (CS) films presented the highest water solubility (~ 60%) and soil degradability (~ 33%). This is consistent with the hydrophilic nature of chitosan, whose amino and hydroxyl groups readily interact with water molecules, facilitating chain hydration and dissolution.

Upon the addition of curcumin, both properties decreased markedly. In terms of water solubility (Fig. 3a), all the curcumin-containing formulations differed significantly from CS (p < 0.05), with no statistically significant differences among the CS-Cur groups themselves. Importantly, the statistical grouping indicated that the incorporation of curcumin, regardless of its concentration, was sufficient to shift the films to a lower-solubility regime. This reduction in solubility suggests that curcumin promotes stronger intermolecular interactions within the matrix, likely through hydrogen bonding and physical associations between chitosan functional groups and the polyphenolic structure of curcumin. As a result, fewer hydrophilic sites remain available to interact with water, and the film network becomes less susceptible to swelling and dissolution, as evidenced by the FTIR spectral shifts observed at 1640, 1560, and 1322 cm⁻^1^ (Fig. 1).

From an application standpoint, lower water solubility is advantageous for edible coatings on high-moisture fruits such as strawberries because it implies greater integrity of the coating during storage (i.e., less wash-off, less thinning, and more persistent surface coverage).

For soil degradability (Fig. 3b), the formulation with the lowest curcumin concentration (CS-Cur-2.5) did not differ significantly from the neat CS film, and a significant reduction was observed only at concentrations of 5 µM and above. The decrease in soil degradability follows a similar trend to that of water solubility and is likely to reflect the same mechanism. It is possible that curcumin induces the formation of a more cross-linked and hydrophobic matrix, contributing to greater resistance to microbial attack and enzymatic degradation in the soil. Importantly, although the incorporation of curcumin reduced soil degradability compared with that of the chitosan film, all formulations still showed measurable mass loss after 31 days of incubation, suggesting that the films retained a degree of biodegradability that may be relevant for sustainable packaging applications. Taken together, these results indicate that curcumin acts as a structural modifier in the chitosan matrix, improving resistance to aqueous dissolution and biological degradation while preserving the biodegradable character inherent to the base polymer.

### Weight loss and soluble solids content (SSC)

**Fig. 4:**
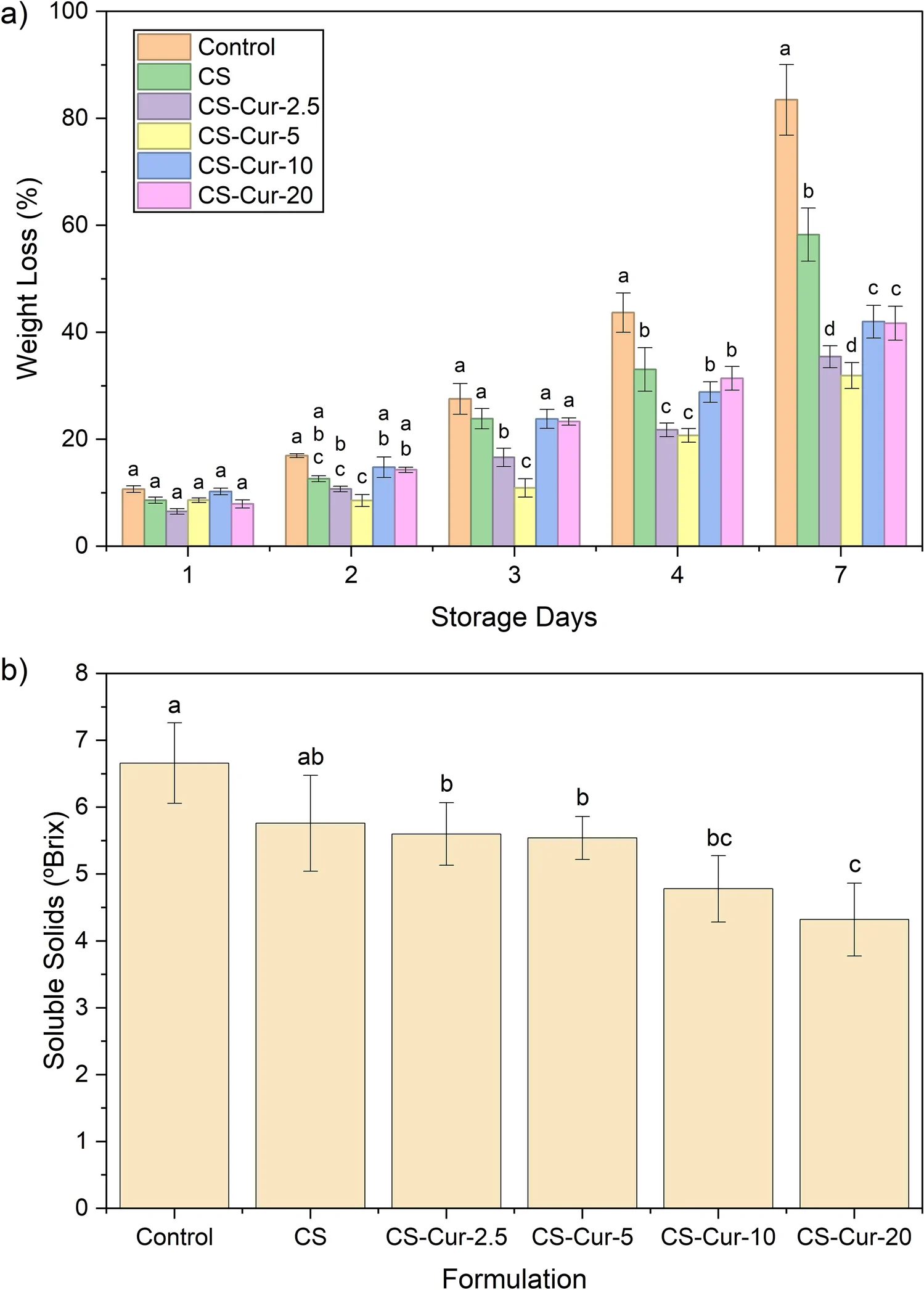
Quality parameters of strawberries under different coating formulations. **a** Weight loss (%) during storage. **b** Soluble solids content (°Brix) after 7 days of storage. The values are the means ± SDs (n = 3 for weight loss; n = 5 for soluble solids). Different letters indicate significant differences among treatments within the same evaluation day (one-way ANOVA followed by Tukey’s HSD, α = 0.05). For weight loss, repeated-measures analysis indicated a significant day × treatment interaction (p < 0.0001)

As shown in Fig. 4, strawberry weight loss increased progressively with storage time, with coated fruit consistently exhibiting lower losses than the uncoated control. Repeated measures analysis revealed a significant interaction between storage day and treatment (p < 0.0001, Table 1S), indicating that the coatings effectively altered the dehydration trajectory. This protective effect became more pronounced in the final stages of storage, particularly after the 3rd day.

The treatment effect was highly significant (p < 0.0001), and the bar classification (Fig. 4a) indicates a protective effect of the coatings against water loss. The uncoated control consistently resulted in the greatest losses over the experimental period, whereas CS (chitosan) reduced dehydration to an intermediate level. This result for the CS film is consistent with the well-established barrier function of chitosan films, which can reduce water vapor transfer by forming a semipermeable layer on the fruit surface and partially modifying gas exchange.

The addition of curcumin to chitosan further reduced weight loss, but the effect was concentration dependent and nonlinear. The CS-Cur-2.5 and CS-Cur-5 samples presented the greatest reduction, whereas the CS-Cur-10 and CS-Cur-20 samples were less effective and presented similar weight losses (Table 2S). This pattern suggests an ideal concentration range in which curcumin improves film performance, likely by increasing hydrophobicity. Mechanistically, higher curcumin concentrations may promote particle aggregation, phase separation, and microstructural defects such as discontinuities or microcracks during drying. A similar behavior has been described by Madian et al. (2023), who observed a dense, spongy-like morphology with a disconnected structure in chitosan films blended with curcumin at higher loadings, attributing this morphological disruption to the strong interaction between curcumin and the chitosan matrix. Furthermore, Zhang et al. (2020) reported that excessive levels of hydrophobic polyphenols can disrupt the continuous polymer network during solvent evaporation. This is further supported by recent findings from Li et al. (2023), who demonstrated that excessive curcumin addition leads to the formation of micro-voids and partial fractures within the chitosan matrix as the film dries, directly resulting in an increased water vapor permeability. These combined observations corroborate our findings regarding the microstructural changes at high curcumin concentrations, which ultimately lead to an increase in water vapor permeability. Furthermore, films with excess curcumin may become more brittle or less adherent to the irregular surface of the strawberry, which can reduce effective coverage and create preferential diffusion pathways. In summary, higher concentrations of the additive do not automatically translate to better barrier performance, as excess curcumin can disrupt matrix continuity.

The interaction effect further shows that the benefits of the coatings are amplified over time. At the beginning of storage, the differences between treatments were modest because cumulative dehydration was still limited. As storage progresses, the vapor pressure gradient between the fruit and the environment persists, and small differences in barrier properties accumulate into large separations.

Another indicator of coating effectiveness is the total soluble solids content (SSC). In strawberries, SSC is associated mainly with low-molecular-weight sugars, such as glucose and fructose, and, to a lesser extent, with organic acids and other soluble constituents (Ali et al., 2022). During postharvest storage, SSC can increase due to metabolic changes related to ripening and water loss, which results in the accumulation of soluble compounds in the pulp. However, SSC is the net result of simultaneous processes, including the metabolism of sugars or acids and the dilution-concentration effects driven by transpiration.

The incorporation of curcumin into the chitosan coatings was associated with a reduction in soluble solids content (SSC) in strawberries after 7 days of storage (Fig. 4b). The SSC decreased from 6.66°Brix (control) to 4.32°Brix (CS-Cur-20), showing a clear concentration-dependent trend and statistically significant differences among the treatments (p < 0.05). This pattern can be discussed in light of the physicochemical properties of the film (Table 1). Increasing the curcumin concentration (2.5–20 µM) produced films with progressively lower moisture contents, ranging from 7.71 ± 0.12% (CS-Cur-2.5) to 6.64 ± 0.10% (CS-Cur-20), alongside a slightly reduced thickness (0.194–0.140 mm), whereas the WVP increased (0.121–0.264 g·mm·m⁻^2^·day⁻^1^·kPa⁻^1^). Thus, the lower SSC in the coated fruit may indicate that the CS-Cur coatings modulated postharvest physiology (e.g., delayed ripening/senescence) or reduced the dehydration-driven solute concentration rather than simply reflecting sugar depletion. Similar coating-mediated moderation of strawberry physicochemical changes during storage has been reported in the literature (Hassan et al., 2025; Silva et al., 2021).

From a mechanistic standpoint, curcumin may alter the coating microstructure and the fruit surface microenvironment (e.g., local gas composition and surface moisture), which can affect the respiration rate, senescence progression, and tissue integrity. This is due to its relatively hydrophobic character and potential interactions with the CS matrix.

While the higher WVP measured for solvent-cast films (Table 1) indicates a lower intrinsic moisture barrier in vitro, this did not result in greater weight loss in the coated fruit, as would be expected from the standardized film measurements alone. The dynamics of moisture loss in vivo are highly complex and extend beyond passive vapor diffusion. While physical interactions at the fruit-coating interface might contribute, the reduced weight loss is likely driven primarily by the biological activity of curcumin. By exerting antioxidant and antimicrobial effects (Dai et al., 2022), curcumin helps maintain tissue integrity and delays senescence (Zhang et al., 2021). This bioactive modulation of fruit physiology likely suppresses natural respiration and transpiration rates, thereby reducing metabolic water loss independently of passive vapor diffusion through the coating (Li et al., 2023). Therefore, the improved moisture retention and the concentration-dependent decrease in SSC observed for the CS-Cur coatings likely reflect a combined effect, where the bioactive preservation of the fruit tissue compensates for the higher intrinsic WVP of the composite films. Comparable effects have been reported in chitosan-based coatings on fruits, where optimized moisture barriers delayed the decrease in SSC by mitigating mass loss and microbially induced breakdown (Hassan et al., 2025; Silva et al., 2021).

While the exact coating uptake on the fruit surface was not quantified, the immersion protocol was strictly standardized to ensure uniform application across all treatments. This methodology is consistent with approaches used in comparable postharvest studies (González-Cuello et al., 2024; Khan et al., 2019). Additionally, the strawberries were purchased from a local market rather than harvested from a controlled production site. This sourcing strategy is commonly adopted in edible coating research (Bağdat and Kahraman Ilıkkan, 2026; Mohamed and Ramaswamy, 2025; Popescu et al., 2022). By incorporating the inherent biological variability regarding pre-harvest factors and maturity, this approach effectively simulates a real-world retail scenario, demonstrating the robustness and practical applicability of the chitosan-curcumin coatings under commercial supply chain conditions.

### Effects of edible coatings on fruit decay, firmness and color

**Fig. 5:**
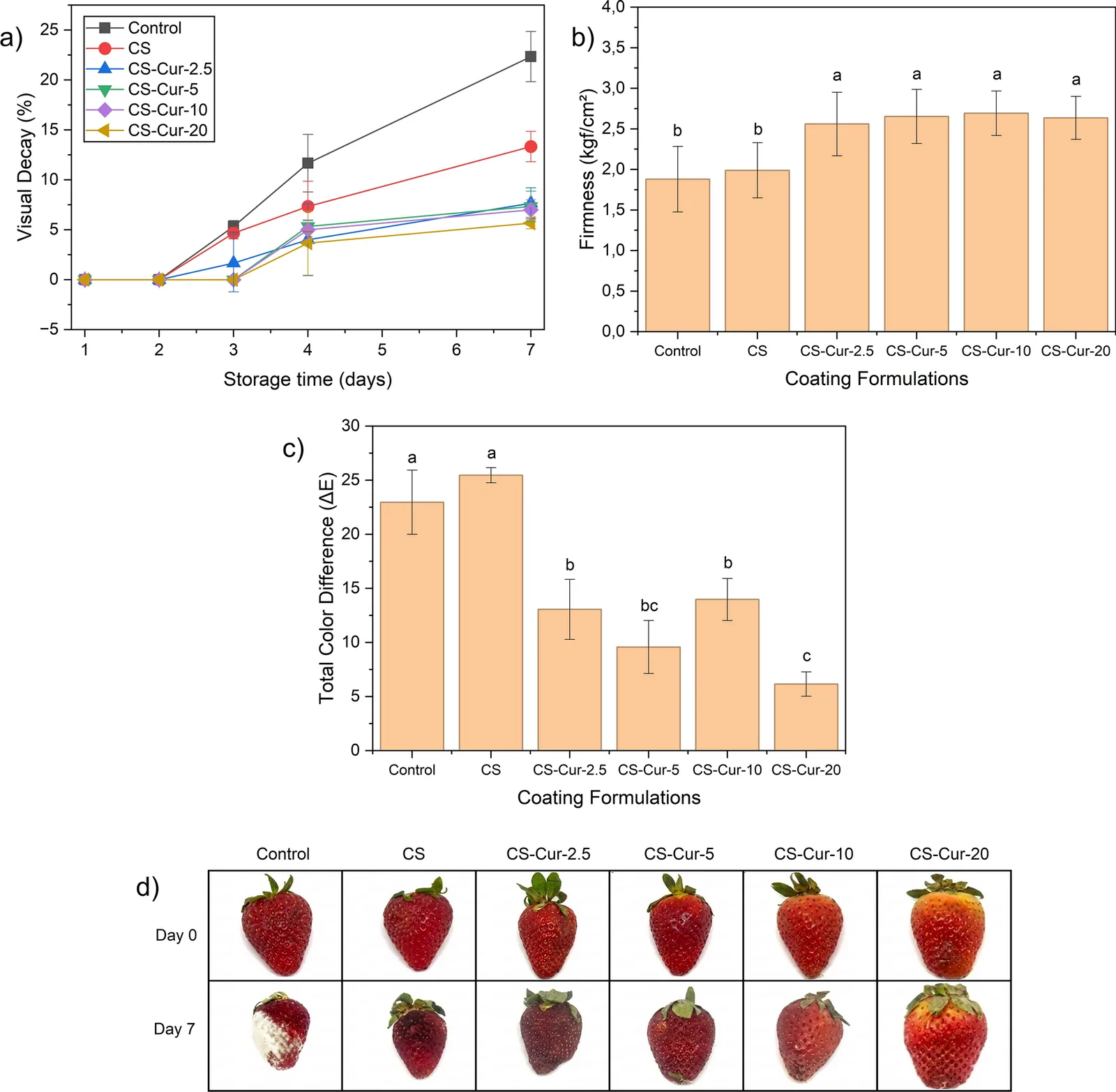
Quality and appearance evaluation of strawberries coated with chitosan (CS) and chitosan–curcumin (CS-Cur) films during storage at 20 °C. **a** Visual decay over 7 days. **b** Firmness after 7 days of storage. **c** Total color difference (ΔE) after 7 days of storage. **d** Representative appearance of strawberries on day 0 and day 7 (brightness adjusted for better visualization). The bars represent the means ± standard deviations (n = 3 for visual decay and color difference; n = 10 for firmness). Different letters above the bars indicate significant differences among treatments (one-way ANOVA followed by Tukey’s post hoc test, p < 0.05)

The effects of chitosan-based coatings on the visual deterioration, firmness, and color variation of strawberries are shown in Fig. 5. Owing to the highly perishable nature of strawberries, all the treatments resulted in a progressive increase in deterioration over the 7-day storage period at 20 °C. However, fruits coated with the curcumin-containing formulations, particularly CS-Cur-10 and CS-Cur-20, consistently presented lower decay rates than did the uncoated control fruits throughout the storage period, with all the curcumin concentrations (2.5–20 µM) showing similar protective efficacy. The neat chitosan (CS) film also provided some degree of protection, which is consistent with the well-documented antimicrobial properties of chitosan, attributed to its polycationic nature and ability to disrupt microbial cell membranes (Duan et al., 2019; Saberi Riseh et al., 2024). The enhanced performance of the curcumin-containing films can be attributed to the broad-spectrum antimicrobial and antifungal activity of curcumin (Hussain et al., 2022), which may act synergistically with chitosan to inhibit the growth of decay-causing pathogens (Cho et al., 2025).

It is worth noting that decay was evaluated based on macroscopic incidence (presence or absence of visible lesions). While this binary visual assessment is a widely accepted method in postharvest studies (Khan et al., 2019; Peretto et al., 2017), we acknowledge that it provides a qualitative measure rather than a quantified severity scale. Nonetheless, this approach was effective to reveal consistent and statistically significant differences among treatments, and the observed trends are in agreement with the objective instrumental data (e.g., firmness and weight loss) discussed below.

Compared with both the uncoated control and the pure CS film, strawberries coated with films containing curcumin maintained significantly greater firmness values (Fig. 5b) after 7 days. No significant differences were observed among the curcumin-containing formulations themselves, suggesting that even the lowest curcumin concentration tested (2.5 µM) was sufficient to produce a meaningful effect on tissue integrity. This outcome may be attributed to the antioxidant and antimicrobial properties of curcumin, which likely mitigate oxidative stress and microbial-driven cell wall degradation during storage (Avendaño-Briseño et al., 2025; El-Saadony et al., 2025). Additionally, the more cohesive and hydrophobic film matrix formed in the presence of curcumin, as evidenced by the physicochemical data, may have contributed to maintaining turgor pressure and, consequently, firmness.

The effects of the chitosan-based coatings on the color of strawberry fruits after 7 days of storage at 20 °C are presented in Fig. 5c and are expressed as the total color difference (ΔE) relative to that of freshly harvested fruits (Table 3S). The uncoated control and the neat chitosan film (CS) presented the highest ΔE values, with no significant difference between them (p > 0.05), indicating that the pure chitosan coating provided little to no protection against color changes under the tested storage conditions. In contrast, all the curcumin-containing formulations significantly reduced ΔE compared with the control and CS groups (p < 0.05), demonstrating a clear beneficial effect of curcumin incorporation on color preservation.

With respect to the curcumin-containing films, the reduction in color variation did not follow a strictly linear trend. The formulations CS-Cur-2.5 and CS-Cur-10 presented intermediate ΔE values and did not differ significantly from each other. Interestingly, the CS-Cur-5 coating performed better than the CS-Cur-10 coating did, reaching a ΔE level statistically similar to that of the highest concentration (CS-Cur-20). Despite this nonmonotonic behavior, CS-Cur-20 presented the lowest overall color variation. These results indicate that the incorporation of curcumin effectively mitigates the chromatic changes associated with strawberry senescence, such as anthocyanin degradation and polyphenol oxidase-driven browning (Silva and Sulaiman, 2022).

Additionally, the more hydrophobic and structurally cohesive film matrix formed at higher curcumin concentrations, as evidenced by the reduced moisture content and water solubility data, may have limited oxygen permeation at the fruit surface, further attenuating oxidative color deterioration.

Furthermore, ultralow curcumin concentrations were selected as a preventive strategy to minimize potential organoleptic impacts, such as strong flavor or intense yellow coloration. As the primary focus of this study was the physicochemical characterization and postharvest performance of the coatings, formal sensory evaluations were not conducted. Future research should incorporate consumer panels to fully validate the sensory acceptability of coated fruits.

Taken together, these results highlight that even at very low concentrations, curcumin significantly enhances the protective capabilities of chitosan films. The developed CS-Cur coatings represent a highly effective, sustainable, and active packaging alternative for extending shelf-life and preserving the quality of highly perishable fresh fruits.

## Supplementary Information

Below is the link to the electronic supplementary material.Supplementary file1 (DOCX 18 KB)
