# Supplementary material for: Ultralow concentrations of curcumin as a structural modifier in chitosan coatings for eco-friendly postharvest preservation of strawberries
Source: Food Sci Biotechnol. 2026 Jul 17;35(11):3295–308. doi: 10.1007/s10068-026-02245-2 (PMC13530090; doi:10.1007/s10068-026-02245-2)
Supplement: Supplementary file 1 [file 10068_2026_2245_MOESM1_ESM.docx]

**Supplementary information**

Table 1S Repeated-measures analysis of strawberry weight loss (%) over storage time.

| **Effect** | **Test** | **Statistic** | **F (df1, df2)** | **p-value** |
| --- | --- | --- | --- | --- |
| Storage day | Pillai’s trace | 0.99978 | 7166.34 (5, 8) | < 0.0001 |
| Storage day × Treatment | Pillai’s trace | 3.24638 | 4.44 (25, 60) | < 0.0001 |
| Treatment (between-subjects) | ANOVA (between-subjects effects) | — | 608.96 (5, 12) | < 0.0001 |

Sphericity was assessed by Mauchly’s test and, when violated, Greenhouse–Geisser correction was applied to within-subject effects.

Table 2S End-point (day 7) and integrated (AUC) weight loss outcomes.

| **Treatment** | **Weight loss at day 7 (%)** | **AUC (%·day)** |
| --- | --- | --- |
| Control | 83.46 ± 6.61^a^ | 267.69 ± 4.51^a^ |
| CS | 58.26 ± 4.95^b^ | 198.54 ± 3.34^b^ |
| CS-Cur-10 | 41.99 ± 3.05^c^ | 169.44 ± 5.20^c^ |
| CS-Cur-20 | 41.69 ± 3.16^c^ | 170.80 ± 4.20^c^ |
| CS-Cur-2.5 | 35.44 ± 2.05^c^ | 130.51 ± 3.85^d^ |
| CS-Cur-5 | 31.91 ± 2.41^c^ | 117.35 ± 1.49^e^ |

Values are mean ± SD (n = 3). Within each column, different letters indicate significant differences among treatments (one-way ANOVA followed by Tukey’s HSD, α = 0.05).

Table 3S CIELab color parameters (L*, a*, b*) of strawberries coated with chitosan (CS) and chitosan–curcumin (CS–Cur) films at day 0 and day 7 of storage at 20 °C, and the corresponding total color difference (ΔE) calculated relative to the initial measurement (day 0).

| **Formulation** | **Day** | **L*** | **a*** | **b*** | **ΔE** |
| --- | --- | --- | --- | --- | --- |
| Control | 0 | 43,59 ± 1,05 | 44,96 ± 2,80 | 21,62 ± 1,58 | 22,96 ± 2,97 |
|  | 7 | 50,08 ± 2,13 | 25,72 ± 3,12 | 11,18 ± 0,5 |  |
| CS | 0 | 41,03 ± 1,21 | 37,99 ± 2,14 | 11,04 ± 0,84 | 25,46 ± 0,70 |
|  | 7 | 34,95 ± 0,70 | 15,08 ± 2,97 | 1,9 ± 0,34 |  |
| CS-Cur-2.5 | 0 | 39,70 ± 0,39 | 26,51 ± 0,77 | 10,48 ± 2,4 | 13,05 ± 2,78 |
|  | 7 | 44,12 ± 0,46 | 16,26 ± 2,82 | 4,01 ± 1,28 |  |
| CS-Cur-5 | 0 | 39,16 ± 3,20 | 33,84 ± 3,27 | 10,47 ± 1,28 | 9,58 ± 2,45 |
|  | 7 | 40,49 ± 1,42 | 24,96 ± 1,88 | 7,86 ± 0,56 |  |
| CS-Cur-10 | 0 | 40,35 ± 0,18 | 42,42 ± 1,48 | 22,75 ± 3,18 | 13,97 ± 1,94 |
|  | 7 | 43,30 ± 1,92 | 25,86 ± 0,99 | 10,51 ± 0,93 |  |
| CS-Cur-20 | 0 | 37,59 ± 3,45 | 40,62 ± 4,5 | 21,56 ± 1,72 | 6,15 ± 1,13 |
|  | 7 | 40,22 ± 4,32 | 36,59 ± 2,61 | 19,32 ± 1,64 |  |

Values are expressed as mean ± standard deviation (n = 3).
